# Human induced pluripotent stem cells integrate, create synapses and extend long axons after spinal cord injury

**DOI:** 10.1111/jcmm.17217

**Published:** 2022-03-08

**Authors:** Nicolas Stoflet Lavoie, Vincent Truong, Dane Malone, Thomas Pengo, Nandadevi Patil, James R. Dutton, Ann M. Parr

**Affiliations:** ^1^ Stem Cell Institute University of Minnesota Minneapolis Minnesota USA; ^2^ Department of Neurosurgery University of Minnesota Minneapolis Minnesota USA; ^3^ University of Minnesota Imaging Center University of Minnesota Minneapolis Minnesota USA; ^4^ Department of Genetics, Cell Biology and Development University of Minnesota Minneapolis Minnesota USA

**Keywords:** differentiation, induced pluripotent stem cells (iPSCs), neuron, oligodendrocytes, spinal cord injury

## Abstract

Numerous interventions have been explored in animal models using cells differentiated from human induced pluripotent stem cells (iPSCs) in the context of neural injury with some success. Our work seeks to transplant cells that are generated from hiPSCs into regionally specific spinal neural progenitor cells (sNPCs) utilizing a novel accelerated differentiation protocol designed for clinical translation. We chose a xenotransplantation model because our laboratory is focused on the behaviour of human cells in order to bring this potential therapy to translation. Cells were transplanted into adult immunodeficient rats after moderate contusion spinal cord injury (SCI). Twelve weeks later, cells derived from the transplanted sNPCs survived and differentiated into neurons and glia that filled the lesion cavity and produced a thoracic spinal cord transcriptional program in vivo. Furthermore, neurogenesis and ionic channel expression were promoted within the adjacent host spinal cord tissue. Transplanted cells displayed robust integration properties including synapse formation and myelination by host oligodendrocytes. Axons from transplanted hiPSC sNPC‐derived cells extended both rostrally and caudally from the SCI transplant site, rostrally approximately 6 cm into supraspinal structures. Thus, iPSC‐derived sNPCs may provide a patient‐specific cell source for patients with SCI that could provide a relay system across the site of injury.

## 1 INTRODUCTION

It has been demonstrated that transplantation of neural progenitor cells (NPCs) into the lesion site after spinal cord injury (SCI) can be beneficial.^1^, ^2^, ^3^, ^4^, ^5^, ^6^ However, many questions remain unanswered regarding which neural cell type is the most beneficial, and whether transplanted neurons have the ability to integrate within the circuitry of the host spinal cord. This question is crucial to addressing chronic SCI because many of the neuroprotective mechanisms shown to be helpful following intervention in subacute SCI will not provide benefit as the lesion stabilizes over time.

NPCs can give rise to three different cell lineages; astrocytes, oligodendrocytes, and neurons.^7^ Neurons have distinct subtypes that are regionally specific to different areas of the brain and spinal cord, and it has been demonstrated that matching regional specificity of the exogenous cells to the injury site is critical to the integration of transplanted neurons. Both spinal cord‐derived (sNPCs) and neocortex‐derived NPCs survive after transplantation into a rodent model of SCI. However, in the spinal cord environment neocortex‐derived NPCs fail to acquire mature neuronal markers, do not integrate and will not extend neurites in vivo. In contrast, sNPCs transplanted into injured spinal cords differentiate into neurons that display mature markers and form functional connections with local neurons.^8^ NPCs derived from foetal, post‐mortem and pluripotent stem cells (ESC and iPSCs) have all been utilized with promising results, however, of these options, only autologous iPSC‐derived NPCs can avoid the need for immune suppressing agents in human clinical trials.^9^ We have previously designed an improved, rapid and clinically relevant method to generate regionally specific spinal neural progenitor cells (sNPCs) from human iPSCs to utilize in transplantation studies^10^ (These iPSC‐sNPCs were originally termed human ventral spinal neural progenitors (hVSNPs) in the previous publication). In this current study, we now evaluate the survival, fate and integration of these iPSC‐sNPCs in a rat model of subacute thoracic contusion injury.

There is evidence that regionally specific human sNPCs transplanted into the spinal cord extend axons over several centimetres into the brain and can potentially provide functional recovery,^8^, ^11^, ^12^ Here, we determine whether our transplanted iPSC‐sNPCs are also capable of forming functional connections with the native rat spinal cord after transplantation and investigate the effects of the sNPCs on the host rat spinal cord environment. This work utilizes a novel protocol designed to generate human iPSC‐sNPCs for clinical transplantation after SCI and investigates whether a relay mechanism is a possible strategy for the treatment of SCI using these cells.

## 2 MATERIALS AND METHODS

### 2.1 Human iPSC culture and sNPC preparation

Human iPSCs were maintained in adherent culture at 37°C in 5% CO_2_ on human vitronectin (rhVTN, AF‐140‐09; PeproTech, Rocky Hill, NJ) in Essential 8 Media (A2858501; Thermo Fisher Scientific, Waltham, MA) and passaged using hypertonic citrate according to our previously published protocol.^13^ The derivation of sNPCs has been previously described [10, Joung et al 2018]. Briefly, cells were passaged at half the density required for maintenance culture (1:6 spit ratio) and cultured for 18–24 h before the Essential 8 media was replaced by Essential 6 media (A1516401; Thermo Fisher Scientific) supplemented with 250 nM LDN‐193189 (S7507; Selleckchem, Houston, TX). Cells were then maintained under these conditions with daily media changes until passage at a 1:10 split ratio onto VTN after day 3. Following passage, the adherent cells were cultured in Essential 6 media supplemented with 250 nM LDN‐193189, 100 nM Retinoic acid (RA, R2625; Sigma‐Aldrich, Billerica, MA) and 3 µM CHIR99021 (4423; Bio‐Techne, Minneapolis, MN) for 8 additional days. The medium was changed daily. By day 11, the cells detach as spherical cell aggregates and were collected and resuspended into Dulbecco's modified Eagle's medium (DMEM) F/12 basal (11039–047; Thermo Fisher Scientific), containing 1× N2 (A13707‐01; Thermo Fisher Scientific), 1× B27 (17504–044; Thermo Fisher Scientific), 100 nM RA and 1 µM smoothened agonist (SAG, 11914; CaymanChem, Ann Arbor, MI). Cell spheres were placed into suspension culture in ultralow attachment plates (3471; Thermo Fisher Scientific) for an additional 6 days with media changes every other day before cryopreservation. For cryopreservation, spheres were resuspended in day 11–17 media with 10% DMSO and cooled in a Mr. Frosty™ Freezing Container (5100–0001, Thermo Scientific™) overnight at −80°C before transferring into liquid nitrogen. The day prior to transplantation, spheres were thawed in day 11–17 media in ultralow attachment plates. On the day of transplantation, sNPCs were dissociated with TrypLE, washed and resuspended at a concentration of 50,000 cells/uL. Cells were kept on ice prior to transplantation. The sNPCs transplanted in this study are the same cells characterized by microarray at the end of Stage 4 on day 17 of the protocol described in detail in Figure 2 of reference 10.

### 2.2 Overview of in vivo study

23 adult female athymic nude (ATN) rats (200–220 g) received a thoracic 8/9 (T8/9) moderate contusion injury. Animals had free access to food and water throughout the study and were housed in a 25°C specific pathogen free facility. All protocols were approved by the Institutional Animal Care and Use Committee (IACUC) at the University of Minnesota. ATN rats were selected because they can readily accept xenografts due to their immune deficiency and our previous experience with RNA‐seq in these rats.^14^

9 days after SCI, rats were randomized to either sNPCs (*n* = 13) or culture medium only (*n* = 10). 3 rats receiving sNPCs were sacrificed at 4 weeks and tissue examined for short‐term cell survival, fate and integration. The remaining 20 rats underwent functional testing as described below. Two rats in each group did not survive; therefore, there were 8 rats per group utilized in the final analysis. These were sacrificed at 12 weeks and tissue examined for cell survival, fate and integration. Immunohistochemistry (IHC) was performed on 5 rats per group and 3 short‐term rats. Additionally, laser microdissection and RNA‐seq were performed (*n* = 3 rats per group) to further examine the cells and the surrounding microenvironment.

### 2.3 Contusion injury and transplantation of sNPCs

A total of 23 adult female ATN rats weighing 200–220 g were used for this study. Rats were anaesthetized, and a laminectomy was performed at the T8/T9 vertebral level. A moderate contusion SCI was made with a 200Kdyn force using the Infinite Horizon Spinal Cord impactor (IH 0400; Precision System and Instrumentation LLC, Fairfax Station, VA). Animals were subcutaneously injected with extended‐release buprenorphine (1.0–1.2 mg/kg) 2–4 h prior to surgery to control pain. Additionally, animals were subcutaneously injected with ceftiofur (1–20 mg/kg) for a period of 5 days after surgery to prevent infection.

Nine days after injury, rats were anaesthetized by inhalation of isoflurane, and the laminectomy site was reopened. A 10‐ul Hamilton syringe with a 32‐gauge needle (0.5 inch long, 30° bevelled tip) was used to inject culture media (10 μl) or sNPCs (10 μl, 50,000 cells/μl, total = 500,000 cells) divided into three separate and equal injection sites at the epicentre and 1 mm rostral and caudal to the epicentre of the lesion. Culture media or cells were injected at the rate of 1 μl/min using a microinjector (Stoelting, Wood Dale, IL, USA). The injection needle was left in situ for five minutes after injection to minimize cellular regurgitation. The surgeon was not blinded to group allocation, but subsequent assessments were performed by blinded examiners.

### 2.4 Assessment of functional recovery

Functional tests were performed before and after the initial injury, after transplantation, and then weekly from injury until sacrifice. Locomotor activity was evaluated using the Basso, Beattie and Bresnahan (BBB) locomotor rating scale,^15^ by two independent blinded examiners. Motor subscores were determined according to the method of.^16^ Ladder walk analysis was recorded and analysed weekly according to a modification of the method of Metz.^17^ All remaining rats were sacrificed at 12 weeks after transplantation.

### 2.5 Tissue harvesting

Rats were fully anaesthetized with intraperitoneal injection of ketamine hydrochloride and transcardially perfused with 4% paraformaldehyde (15714‐S; Electron Microscopy Sciences, Hatfield, PA, USA) in 0.1 M phosphate‐buffered saline (PBS), pH 7.4. Spinal cords were removed and post‐fixed overnight in the same fixative solution, then immersed in sucrose (30% w/v) and washed with PBS. A segment of the spinal cord 1.0 cm in length encompassing the injury site was removed and embedded in Tissue‐Tek OCT embedding compound (VWR, Mississauga, ON, Canada). The tissue was sectioned in the sagittal plane at 10 µm intervals using a Leica CM3050 S cryostat.

### 2.6 Cell counting

To quantify the number of surviving transplanted cells in the spinal cord, 15 sections were cut from each rat spinal cord in the parasagittal plane at 10 μm thickness, 160 μm apart, and all cells in the 15 sections were counted. Transplanted cells expressing human nuclear antigen, with typical cell morphology and clearly delineated cell borders, were counted using Image J software from Fiji (v.1.45) (NIH; Bethesda, MD). The cell counts were then adjusted using the Abercrombie method.^18^ This number was expressed as a percentage of cells surviving with the number of live cells injected as the denominator (500,000 cells per rat) in rats that were sacrificed 12 weeks after transplantation.

### 2.7 Cavitation analysis

Cavitation analysis was performed for the long‐term rats only (*n* = 5 rats per group). To analyse cavitation, every eighth section was processed with Nissl & Eosin Y staining. Ten sections per rat (rostro‐caudally 1.6 mm from the epicentre of cavity) were imaged on a Leica DMi8 inverted microscope. To compare cavity area between groups, a modified protocol which combines Nissl and Eosin Y staining was developed. The area of maximum cavitation (epicentre) of each section was traced using *Image J* software from Fiji (v.1.45) (NIH; Bethesda, MA). The measurements obtained were used to generate values for the cavity volume for each of the cords from each treatment group. The total cavity volume and total spinal cord volume were calculated using the Cavalieri method,^19^ and the percentage cavitation determined.

### 2.8 Immunohistochemistry (IHC)

IHC analysis was performed for both the short‐term (n=3 rats per group) and the long‐term rats (*n* = 5 per group). For fluorescence immunostaining, antibodies were utilized to identify the transplanted cells with either human nuclear antigen (hNA, MAB1281; 1:250; Millipore, Billerica, MA) or human cytoplasmic marker (Stem121, AB‐121‐U‐050; 1:250; SC Proven, Newark, CA). We used two different antibodies because one stains nuclei whereas the other stains cytoplasm. When counting cells, a nuclear stain can be more useful; however, a cytoplasmic stain can also demonstrate dendrites and axons. In any given assay, only one of the two antibodies was utilized. Other IHC antibodies were utilized for cell identification including Ki‐67 (ab15580; 1:250; Abcam, Cambridge, UK) for dividing cells, neuronal nuclei (NeuN, EPR12763; 1:1000; Abcam) for mature neurons, microtubule‐associated protein 2 (MAP2, ab32454; 1:200; Abcam) for neurons and their dendrites, synaptophysin for synapses (Syn, 101–004; 1:500; Synaptic Systems, Gottingen, Germany), glial fibrillary acidic protein for astrocytes (GFAP, Z0334; 1:500; Dako, Santa Clara, California), adenomatous polyposis coli for mature oligodendrocytes (APC, ab15270; 1:100; Abcam), nestin for neural stem cells (ABD69; 1:250; Millipore), myelin basic protein (MBP, AB40390; 1:250; Abcam) and terminal deoxynucleotidyl transferase dUTP nick end labelling for cell apoptosis (C10617; Thermo Fisher Scientific). Sections were incubated with secondary antibody conjugated to Alexa Fluor 488, 555 or 647 (A‐31572, A‐21206, A‐21432, A‐11055, A‐31570 and A‐21202; 1:500; Thermo Fisher Scientific). Negative controls were obtained by omission of the primary antibody. Positive controls were also included. DAPI (62248; 1:1000; Thermo Fisher Scientific) was utilized to counterstain DNA. The proportion of transplanted cells that were double‐labelled was calculated for each rat spinal cord examined.

### 2.9 Tissue clearing

CLARITY^20^ tissue clearing was performed with an X‐CLARITY system (Logos Biosystems, Annandale, VA) on *n* = 3 rat brains in the transplant group and tissue imaged at 20× 0.95 NA with a ribbon confocal microscope (RS‐G4, Caliber I.D., Andover, MA). For intact brain tissue that underwent clearing, clearing solution was circulated continuously at 37°C, 1.5 A current, and 30 rpm pump speed for 12 h. After samples underwent active electrophoretic clearing, they were subjected to fluorescence immunostaining with Stem121 primary antibody and Alexa Fluor 488 secondary labelling before imaging.

### 2.10 Laser dissection microscopy (LDM) and RNA preparation

LDM was performed on long‐term rats only (*n* = 3 rats per group). Histological staining for Laser Microdissection was performed as adapted from.^21^ Briefly, slides were fixed with 95% ethanol, and stained for 20–25 s in 70% commercial eosin Y at 0.5% in ethanol (HT110232‐1; Sigma‐Aldrich) and 30% cresyl violet at 4% w/v (C5042; Sigma‐Aldrich) in ethanol. Slides were then rinsed and dehydrated in 100% ethanol, then dehydrated in xylene for 3 min and dried in a vacuum desiccator. Sections were dissected, and RNA was prepared by adding 200 µl 1‐Bromo‐3‐chloropropane (AC106686; Acros Organics, Fair Lawn, NJ) to Trizol and incubating the sample for 15 min on ice. After centrifugation, the aqueous phase was aliquoted and 250 µl of isopropanol (BP‐2618; Thermo Fisher Scientific) was added. The sample was re‐incubated for 10 min then centrifuged at 12,000 g for 10 min. Supernatant was removed, and the pellet was washed with 0.5 ml of RNAse free 70% ethanol. The sample was then centrifuged at 7500 g and 4°C temperature for 10 min, and the supernatant was removed. The pellet was dissolved in 40 µl of RNAse free H_2_O and frozen on dry ice. The samples were collected and sent to the University of Minnesota Genomics Core for quality control and library preparation.

### 2.11 Transcriptomic analysis

Samples for RNA‐seq analysis included in vitro sNPCs, in vivo transplanted sNPCs prepared using LDM and host spinal cord microenvironment prepared using LDM. RNA samples were prepared in triplicate for RNA‐sequencing. Briefly, total RNA samples were quantified using the RiboGreen fluorometry assay and RNA integrity was confirmed using capillary electrophoresis. Integrity of submitted RNA was also assessed using Nanodrop and Bioagilent Analyzer 2100 (RINs ranged from 8.4 to 10.0). Total DNA contamination was quantified using the Picogreen fluorometry assay. Libraries were generated from 250 ng of total RNA. Polyadenylated coding mRNA in each sample was isolated and reverse transcribed using random primers. The resulting paired‐end cDNA libraries were subsequently sequenced using an Illumina HiSeq 2500. For each sample, at least 20 million paired‐end reads of 50 base pairs were performed in four lanes. FASTQ files for each sample were combined, and raw sequences were analysed using a customized pipeline developed and maintained by the Minnesota Supercomputing Institute (gopher‐pipelines; https://bitbucket.org/jgarbe/gopher‐pipelines/overview).^22^ Briefly, quality controls were performed on each FASTQ files using FastQC (v0.11.5) before and after trimming with Trimmomatic (v.033). Trimmed sequences were aligned using HISAT2 (v2.02). Transcript abundance was then estimated and differential gene expression was determined using Cufflinks (v2.2.1). Read counts generated by subread were filtered and the remaining reads normalized and log transformed using *edgeR*. Heat maps were generated using the log‐transformed values with *pheatmap* package. Hierarchical clustering was performed using Euclidean distances and average linkage clustering method, and principal component analysis was performed in R using *prcomp* and visualized using *ggplot2*.

### 2.12 qPCR probe design

Total cellular RNA was isolated with TRIzol (Invitrogen) extraction. Reverse transcription was performed using ProtoScript First Strand cDNA Synthesis kit (New England Biolabs). The qPCR reactions were carried out using PerfeCTa SYBR Green SuperMix following the manufacturer's protocols. Relative levels of mRNA of interest were calculated based on ΔCt values and subsequent normalization to ACTIN mRNA levels. The following qPCR primers were in these assays:

NEUROD6 Forward 5’ TACATCTGCGCAGCCAATCT 3’.

NEUROD6 Reverse 5’ GGGGAGGTGAATGACCACTG 3’.

OLIG1 Forward 5’ TGTCGCAGAGAGTTTTCGCT 3’.

OLIG1 Reverse 5’ ATGCAAGGCGGTTGGTTTTC 3’.

CACNG3 Forward 5’ CCGAATATCTCCTGCGAGCTG 3’.

CACNG3 Reverse 5’ CGCGCTGAGAATGACGTTG 3’.

HCN2 Forward 5’ CGTACAGCGACTTCAGGTTCTA 3’.

HCN2 Reverse 5’ GTCCGAGACCACGTTGAACA 3’.

LYNX1 Forward 5’ ATCCCCTGGGGTTGCAG 3’.

LYNX1 Reverse 5’ CGCACAGAGGATCCAACTCA 3’.

ATP1A3 Forward 5’ TCATGTAGGGGAGCAGGCAT 3’.

ATP1A3 Reverse 5’ TCATCTTTCTTGTCCGTGGCT 3’.

SOX10 Forward 5’ CTCTGGAGGCTGCTGAACG 3’.

SOX10 Reverse 5’ CGGCCTTCCCGTTCTTCC 3’.

SLC32A1 Forward 5’ TCCATTATCAGCGAGGCAGC 3’.

SLC32A1 Reverse 5’ ACGAACATGCCCTGGATGG 3’.

ACTIN Forward 5’ GAGCACAGAGCCTCGCCTTT 3’.

ACTIN Reverse 5’ ATCATCATCCATGGTGAGCTGG 3’.

### 2.13 Data analysis

All quantitative data are presented as mean ± SEM. Open field locomotor scores (BBB) for the hind limbs from the same group were averaged to yield one score for each time point. BBB scores, motor subscores and ladderwalk data were compared using repeated measures analysis of variance (ANOVA) comparing control versus experimental group over time, with time taken as a repeated measure. Post hoc analysis was performed using the Bonferroni test where indicated. Cavitation volume, as well as transplant survival, differentiation, proliferation and integration, was compared utilizing a Student t‐test. *Nikon Elements* DenoiseAi (RRID:SCR_014329) algorithm was utilized to reduce background in tissue cleared samples. *Agave* ray tracing software was utilized for 3D visualizations in Figure 4G,I. In all cases, analysis of quantitative data was performed for overall significance with null hypotheses rejected at the level of *p *< 0.05. For the RNA‐seq data, two‐sided Student's *t*‐test was performed to assess the differential expression with a global false discovery rate set at 0.5. Both the size of transcript and the number of aligned reads for genes were normalized using CuffNorm ‘Fragments per Kilo base of transcript per Million mapped reads (FPKM)’, which indicates the relative expression level.

## 3 RESULTS

### 3.1 Survival and integration of human iPSC‐sNPCs transplanted into the injured rat spinal cord

**FIGURE 1:**
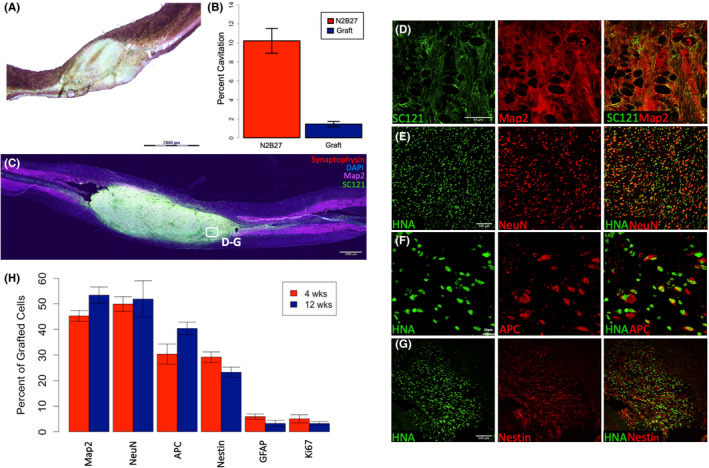
Transplanted Human iPSC‐Derived sNPCs Fill the Lesion Cavity and Express Mature Neural Markers. (A) Human iPSC‐derived sNPCs were transplanted into T8 contusion SCI. Sagittal sections of Eosin Y/Nissl staining. (B) Cavitation analysis of the contusion site and transplanted cells in 1A. N2B27 is a media‐only control. (C) Immunolabelling of transplanted cells filling the lesion cavity. Rostral is left and caudal is right. Note: D‐G are representative examples. (D–G) Expression of neural markers Nestin, NeuN, Map2 and APC in transplanted human cells (HNA/SC121) twelve weeks post‐transplantation. (H) Quantification of expression of neural markers at 4 and 12 weeks post‐transplantation. Both Ki‐67 and GFAP expression are less than 5% after twelve weeks and approximately 90% of transplanted human cells are expressing mature neural markers. Data are represented as mean ± SEM

At 12 weeks post‐transplantation, cells derived from the transplanted human iPSC‐sNPCs^10^ (Figure S1) were found to fill the lesion cavity resulting from the contusive SCI (Figure 1A). In 6 rats, the transplanted cells completely filled the cavities and in 2 rats the transplanted cells almost entirely filled the cavities. Cavity volumes were significantly reduced in the sNPC group compared to control rats that had no cells transplanted (Figure 1B). Cell survival was estimated to be 70.03% ± 9.23%.

In tissue harvested 4 weeks after transplantation approximately half of the cells at the injury sites derived from the transplanted iPSC‐sNPCs expressed neuronal markers (Figure 1C‐G). The majority of transplanted cells expressed the mature neuronal markers MAP2 and NeuN (MAP2 – 45.2% ± 2.1%, NeuN – 49.9% ± 2.8%). After 12 weeks, similar numbers of transplanted cells displayed neuronal markers (MAP2 – 53.4% ± 3.1%, NeuN – 51.8% ± 7.1%) (Figure 1D,E,H). In contrast, fewer transplanted cells expressed the mature oligodendrocyte marker APC at 4 weeks (30.3% ± 3.9%) compared to 40.3% ± 2.4% at 12 weeks (*p* < 0.002) (Figure 1F,H). 4.2% ± 1.0% of transplanted cells expressed the mature astrocyte marker GFAP at 4 weeks compared with 3.5% ± 1.2% at 12 weeks (*p* < 0.005) indicating a small contribution of astrocytes from the transplanted cells in these experiments (Figure 1H).

In addition, 29.1% ± ± 2.0% of the transplanted cells expressed the immature marker nestin at 4 weeks and 23.2% ± 2.0% still expressed nestin at 12 weeks (*p* < 0.001) (Figure 1G,H). Proliferation in the transplanted cells was low, indicated by Ki‐67 expression in only 6.3% ± 1.6% of HNA expressing cells at 4 weeks and 3.2% ± 0.72% at 12 weeks, with a trend towards decreasing proliferation over time. TUNEL staining was performed to determine whether there was ongoing apoptotic cell death, and less that 5% of the transplanted cells showed this at both 4 and 12 weeks (data not shown). No teratomas or other abnormal tissue growth was observed at any time point in this study.

### 3.2 Transplanted human iPSC‐sNPCs demonstrate a spinal cord transcriptional program in vivo

**FIGURE 2:**
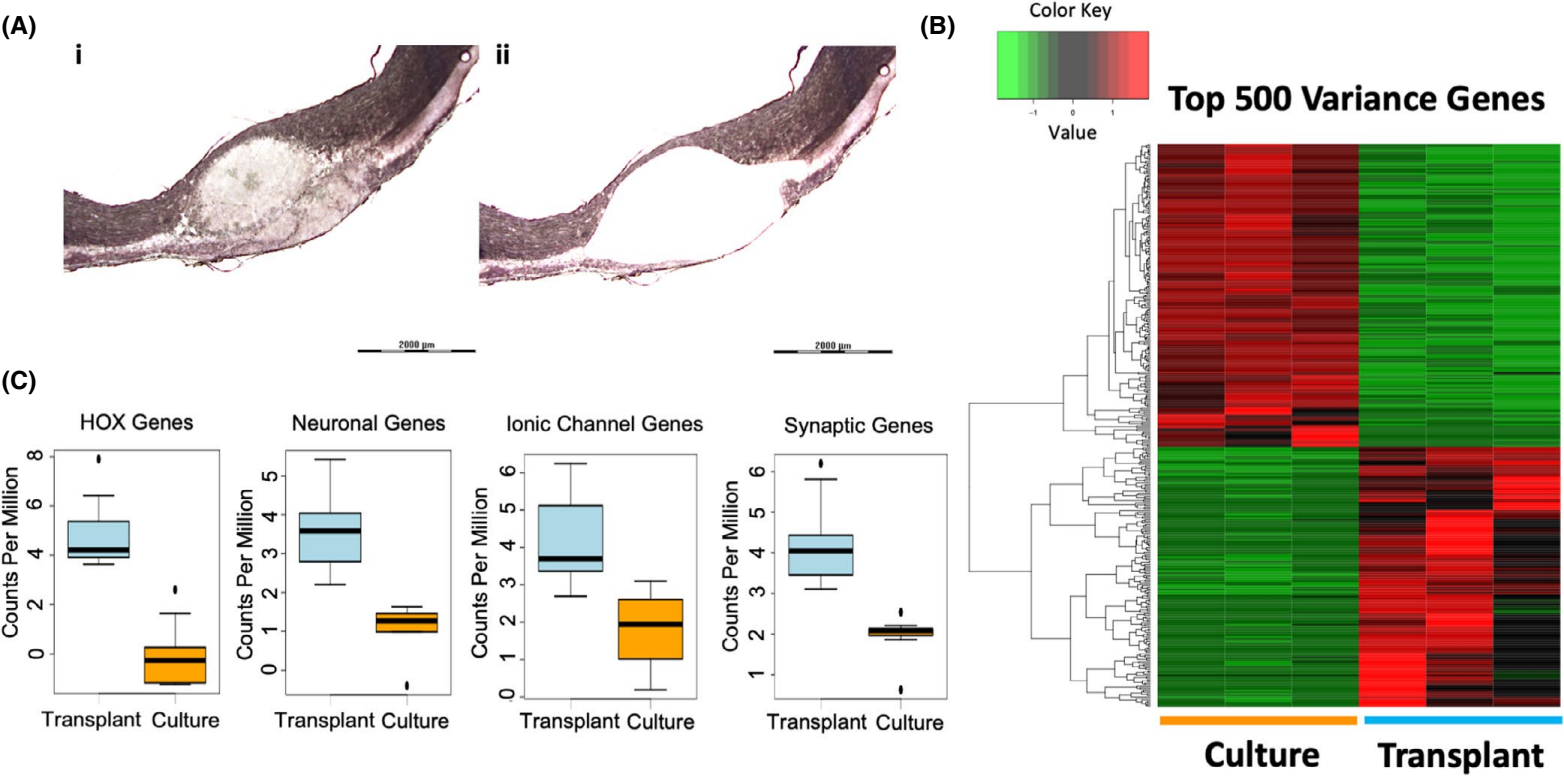
Transplanted sNPCs mature in a contused rat cord. (A_i‐ii_) Area of cell transplantation extracted via laser microdissection for RNA‐sequencing after Eosin Y/Nissl staining. Laser dissection of approximately 200 μm outside of the cell transplantation area for examining the impact of treating contused spinal cords with sNPCs. (B) Unsupervised hierarchical clustering showing distinct separation between transplanted cells and cultured cells. Read counts were normalized and log transformed. Hierarchical clustering was performed using Euclidean distances and average linkage clustering method. The first three and last three columns represent their respective replicates. (C) Expression profile alterations between HOX genes, neuronal genes, ionic channel genes and synaptic genes, respectively, between grafted and cultured sNPCs. The boxes show the 25th–75th percentile range, and the centre mark is the median. Whiskers show 1.5 times IQR from the 25th or 75th percentile values

We utilized RNA‐sequencing (RNA‐seq) to analyse molecular differences between the in vitro cell identity of the sNPCs and expression patterns in these cells 12 weeks after transplantation in vivo. We utilized laser microdissection to specifically isolate cells from the transplantation site (Figure 2A). Unsupervised hierarchical clustering revealed distinct separation between the two groups (Figure 2B). The top 500 differentially expressed genes revealed that transplanted sNPCs developed a regional identity associated with the thoracic spinal cord after transplantation, including HOXA7 and HOXB9 (top 20 differentially expressed genes) (Figure 2C, left). The majority of the contents in the cyst cavities stained for SC121 after 12 weeks which is suggestive of viable tissue. Furthermore, we performed TUNEL staining to identify apoptosis and this was very low.

Analysis of transcription data indicated that the transplanted sNPCs matured primarily into either neurons (NRN1, CEND1, CALY, FBX02, NEUROD4, CDK5R2, NEUROG1, NEUROD6, FOXN4) (Figure 2C, middle left and Figure S2), or oligodendrocytes (OLIG1, SOX10, MOG, MBP, CLDN11) supporting the immunohistochemistry results. Additionally, cell progeny from the transplanted sNPCs differentially expressed a complement of ionic and neurochemical receptors suggesting that sNPCs may be functionally active. We are currently obtaining electrophysiological data to further support this finding. Specifically, there were increases in calcium channel expression: (CACNG3, CACNG2, CACNG7, CALB2), sodium channel expression: (HCN2, SLC6A9, SLC6A17), nicotinic acetylcholine receptor expression: (LYNX1),^23^ GABA receptor expression: (SLC32A1, GABRA5, GABRA3, GABRQ), potassium channel expression: (KCNJ3, KCNJ16, KCTD16, KCTD8, KCNJ10), sodium/potassium pump expression: (SLC24A3, ATP1A3), serotonin receptor expression: (HTR2C) and glutamatergic receptor expression: (GRID1, GRIK4, GRIN3A, SLC1A1, GRIN2D). These data also indicate that there are at least two separate pools of neurons, namely GABAergic and glutamatergic neurons, reflecting the potential for both inhibitory and excitatory signalling. Additionally, we found increased expression of genes associated with axonal extension such as PLXNB3, SLITRK1, GPR12 and NEXMIF. (Figure 2C, middle right). Finally, transplanted sNPCs also upregulated synaptic genes DLGAP3, SNPH, SYNGR3, SYT5, RIMS4, SYT3, SYBU, SYN1, SNCB and SYT13 (Figure 2C, right) further suggesting transplanted neurons may be functionally active and that transplanted neurons may be in a state of higher plasticity.^24^, ^25^, ^26^

### 3.3 Alterations in transcription occurred in the human iPSC‐sNPC‐treated rat spinal cord

**FIGURE 3:**
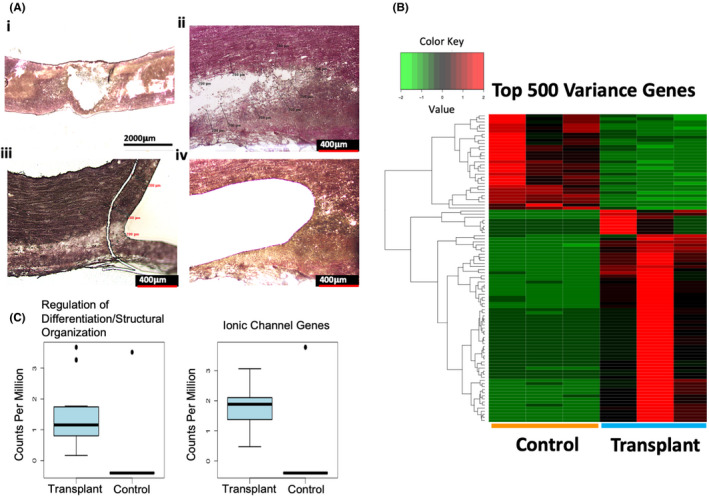
Transplanted sNPCs alter the host transcriptome at the transplantation site. (A_i_) Eosin Y/Nissl staining of untreated contused spinal cord twelve weeks after injury. (A_ii_ and A_iv_) Higher magnification image of the area laser microdissected in untreated contused spinal cords. (A_iii_) Higher magnification image of the area laser microdissected in the treated contused spinal cord. The cavity area was approximated and 200 μm outside the edge of the cavity area was extracted to compare transcriptome profiles with the sNPC‐treated cord. (B) Unsupervised hierarchical clustering reveals distinct separation between host spinal cords with cell grafts and untreated spinal cords. Read counts were normalized and log transformed. Hierarchical clustering was performed using Euclidean distances and average linkage clustering method. The first three and last three columns represent their respective replicates. (C) Expression profile alterations between genes associated with regulation of differentiation/structural organization and ionic channel genes, respectively, between grafted and untreated spinal cords. The boxes show the 25th–75th percentile range, and the centre mark is the median. Whiskers show 1.5 times IQR from the 25th or 75th percentile values

We next explored the molecular alterations in the host spinal cord immediately adjacent to transplanted sNPCs, in order to investigate the effects of the cell transplants on the host injury microenvironment. Twelve weeks after cell transplantation, we excised 200µm of host tissue adjacent to the cell transplant using laser microdissection and subsequently sequenced mRNA isolated from this area (Figure 3A,B). The host tissue immediately adjacent to sNPC transplants was compared with the same area in the tissue surrounding the injury cavity area in rats receiving cell medium only injection. In host spinal cord tissue adjacent to transplanted cells, we observed increases in collagen expression (COL6A4P1) compared to tissue from injured rats without cell transplantation (Figure 3C, left). We also observed increases in factors associated with stem/progenitor cell regulation (TP63, ADAM28, MUC4). Additionally, we found that there were alterations in sodium and calcium ion channel expression (SCN11A, TRPM5, CACNA1F) in the sNPC‐treated host spinal cord directly adjacent to the transplant site when compared to the medium only injured spinal cord (Figure 3C, right). This indicates that transplantation of sNPCs alters the host injury environment after transplantation.

### 3.4 Transplanted human iPSC‐sNPCs generated neurons that extended axons both rostrally and caudally from the transplant site and formed synapses

**FIGURE 4:**
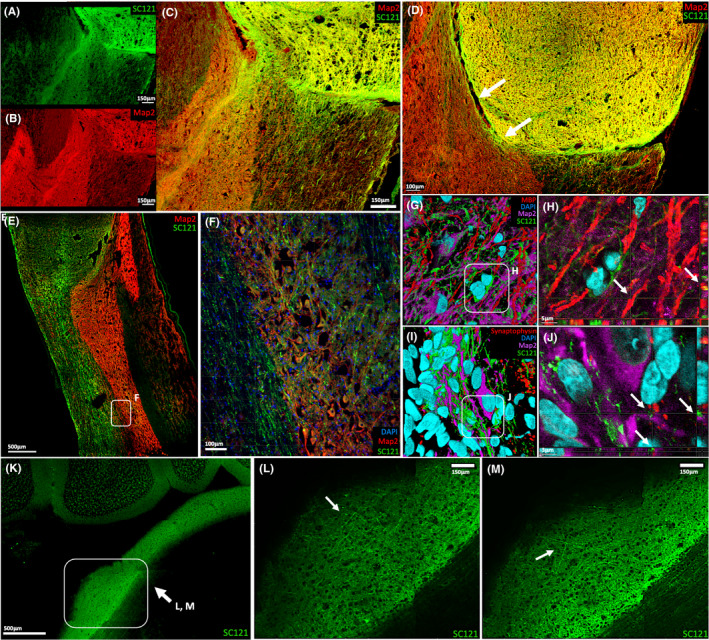
Axonal Extension and Connectivity of iPSC‐derived sNPCs. (A‐C) SC121 and MAP2 staining identify mature transplant‐derived axons projecting into the host spinal cord. (D) SC121 and MAP2 staining indicate that successful axonal projection from the transplant into the host requires cell‐to‐cell contact. (E–F) SC121 and MAP2 staining also demonstrate that these axons extended in a rostral‐caudal direction in the rat spinal cord white matter, with axonal projections occasionally branching off and synapsing on host grey matter. (G‐H) SC121/MBP‐positive cells were located in close proximity and linearly aligned to host MAP2‐positive neurons, suggesting transplanted oligodendrocytes are myelinating host neurons. Arrows indicate 3D rendering of SC121/MBP‐positive cells in linear alignment with host MAP2‐positive cells. (I–J) Host MAP2‐positive neuron surrounded by SC121‐human synaptophysin‐positive puncta, suggests the establishment of synaptic contacts between the transplant and the host. Arrows indicate 3D rendering of a host MAP2‐positive neuron in close proximity to SC121‐human synaptophysin‐positive puncta. (K–M) Tissue clearing reveals human (SC121) axons project up to 6cm from the transplantation site into distinct supraspinal structures within the host, such as the pons

The majority of cell bodies derived from the transplanted sNPCs were located at or adjacent to the injection sites 12 weeks after injury, with processes extending away from the site of injury. These human cells extended large numbers of axons both rostrally and caudally from the lesion site into the host rat spinal cord (Figure 4A,B). We found that most of the transplanted cells (> 90%) expressed mature markers in the cell soma. These cell soma expressed SC121 antibody, as well as the neuronal dendrites and axons. Co‐localization with MAP2 was assessed 12 weeks post‐transplantation to identify neurons and their dendrites. Approximately 15% of the projections out of the transplant expressed MAP2 (Figure 4A‐C). These dendrites typically ended in the grey matter. These findings agree with previously published reports^11^, ^27^ in which many cell soma within the transplants express mature neuronal markers, and these extend MAP2+ dendrites into the grey matter in the vicinity of the graft (<2 mm).

Additionally, our data support previous findings^11^, ^28^ that transplanted cells require direct cell‐to‐cell contact with the host in order to project axons (Figure 4D). We found that the majority of these human axons extended in parallel within the white matter in a rostro‐caudal direction (Figure 4E,F) extending 6 cm, nearly the entire length of the spinal cord into discrete supraspinal structures (Figure 4K–M, and Figure S3). Furthermore, synaptophysin was located adjacent to human axons and host cells within the host spinal cord, suggesting the ability of transplanted human cells to contact and potentially communicate with the host spinal cord (Figure 4I,J). In addition, host oligodendrocytes were directly adjacent to transplanted cell axonal projections, indicating myelination of the transplanted neurons by the host (Figure 4G,H). Furthermore, host myelin basic protein (MBP) was located in close proximity to SC121/MAP2‐positive neurons, suggesting that axonal extension in transplanted cells was not inhibited by host myelin in either the grey matter or white matter. Despite these promising findings, no functional recovery was observed in these experiments.

## 4 DISCUSSION

There has been renewed interest in the role of new neurons generated following NPC transplantation at CNS injury sites after recent publications have demonstrated that they can produce functional benefit in both rodent and primate models.^29^, ^30^ Earlier studies exploring the role of neurons after neural cell transplantation demonstrated very poor survival of neural cell transplants in general, and almost no survival/differentiation into neurons.^31^, ^32^, ^33^, ^34^ In this study transplanting human iPSC‐sNPCs into ATN rats, we have found that not only do the transplanted cells survive, but they can differentiate into mature neural cell types, fill the contused area of the spinal cord and have the ability to extend axons long distances within the central nervous system. These new findings support similar recent published reports of high levels of transplanted cell survival and neural differentiation in animal models and highlight the importance of the potential of autologous cell therapy, which will not require immune suppression, but is potentially achievable through the use of iPSC technology.

Using global transcriptome profiling, we identified expression of genes in the progeny of the transplanted cells associated with both neuronal and oligodendrocytic maturation and found these to be upregulated after transplantation. Interestingly, we found increased expression of FBX02, which suggests a population of transplanted neurons have committed to a V2b lineage. This finding also supports previous reports that the injury microenvironment may alter terminal cell fates, as our cells primarily differentiate into a V2a lineage in vitro. We also observed gene expression patterns consistent with oligodendrocyte maturation, which included CLDN11, OLIG1, SOX10, MOG and MBP, supporting our immunohistochemical analysis of human cells in the transplanted cord sections and indicating that the oligodendrocytes differentiated from the transplanted cells may mature sufficiently to produce myelin. In addition, our analysis indicates that the sNPC transplants differentially express a complement of ionic and neurochemical receptors suggesting that neurons may mature and become functionally active after transplantation. We also noted expression profiles within the graft that were correlated with the impressive axonal extension of transplanted cells within the host. Specifically, GPR12 expression, observed to increase in our study, is associated with neurite outgrowth and blocking of myelin inhibition in neurons and could serve as a potential therapeutic target.

Alterations in host spinal cord transcriptomic profiles induced by transplanted sNPCs immediately adjacent to the injury site have not been previously investigated. We employed transcriptomic analysis of the host tissue microenvironment surrounding the sNPC transplants and compared the expression patterns from injured spinal cords that did not receive cell transplantation. We found that transplantation of sNPCs led to increased expression of ionic channel proteins within the host (SCN11A, TRPM5, CACNA1F) and increased expression of factors associated with stem/progenitor cell regulation (TP63, ADAM28, MUC4). This finding supports the concept that the transplanted cells may promote the expression of neurogenic factors in the host spinal cord that influence cellular differentiation and regulation of stem/progenitor cells. Additionally, our findings support recent evidence that transplantation of neural progenitor cells enables the host corticospinal tract to maintain a pro‐regenerative transcriptome after injury.^35^ However, this remains speculative and further research is needed to support our hypotheses.

In 2014, Lu et al. found that iPSC‐derived NPC transplants in the rat spinal cord generated neurons that extended tens of thousands of axons spanning the entire length of the host CNS, marking one of the first major studies showing evidence of in vivo long‐distance axonal growth from cells derived by iPSC technology.^11^ In 2018, Dell’Anno et al. corroborated these findings by showing that transplanted post‐mortem‐derived sNPCs can also develop into neurons with axons that extend over the entire length of the rat spinal cord. Using our human iPSC‐sNPCs, we also observed similar differentiation and long‐distance axon extension. We found that the presence of a ‘gap’ between the transplant and the host tissue results in a failure of these axons to extend across the lesion border. However, when cell‐to‐cell contact between transplanted cells and host tissue was present, dendrites and axons were seen to extend into the native rat spinal cord and some terminated within the spinal cord in the grey matter. Furthermore, there was evidence of synapse formation at the end of these axons, supporting the concept of functional integration^36^, ^37^, ^38^, ^39^, ^40^, ^41^, ^42^, ^43^ and this corresponds with the recent report by Ceto et al.^44^ showing that dissociated murine embryological sNPC grafts can form extensive networks when transplanted into a mouse spinal cord injury model. Our work helps advance these findings towards the clinic by utilizing a regionalized source of human neural progenitor cells suitable for potential autologous transplantation.

In this study, we did not find functional benefit, and other studies have produced mixed results. There are several possible explanations for this. It is possible that the studies have not been carried out long enough for the cells to fully mature, form appropriate functional synapses, and provide benefit. It has been shown that it could take many months, even more than a year, for human progenitor cells to fully differentiate following transplantation.^11^, ^12^, ^45^, ^46^ In these experiments, we did find that the majority of cells (~90%) expressed mature neural markers 12 weeks post‐transplantation; however, we do not know what further capabilities a mature neuron might have in terms of neuroplasticity. Another possible explanation is that the study was underpowered to detect any subtle differences in locomotor change or that the functional synapses require reinforcement through activation such as that provided by physical therapy or electrical stimulation.

## CONFLICTS OF INTEREST

The authors declare no competing interests.

## AUTHOR CONTRIBUTION

**Nicolas Stoflet Lavoie:** Formal analysis (equal); Writing – review & editing (equal). **Vincent Truong:** Conceptualization (equal); Data curation (equal). **Dane Malone:** Writing – original draft (equal). **Thomas Pengo:** Resources (equal); Software (equal); Visualization (equal). **Nandadevi Patil:** Data curation (equal); Methodology (equal); Validation (equal). **James R. Dutton:** Conceptualization (equal); Project administration (equal); Supervision (equal). **Ann M. Parr:** Conceptualization (equal); Funding acquisition (equal); Methodology (equal); Project administration (equal); Supervision (equal); Writing – review & editing (equal).

## CONTACT FOR REAGENT AND RESOURCE SHARING

Further information and requests for resources and reagents should be directed to and will be fulfilled by the Lead Contact, Ann M. Parr (amparr@umn.edu).

## Supporting information

Figure S1

Figure S2

Figure S3

## Data Availability

The data sets and code utilized in this study are available upon request.
