# Supplementary figures and images for: Human induced pluripotent stem cells integrate, create synapses and extend long axons after spinal cord injury
Source: J Cell Mol Med. 2022 Mar 8;26(7):1932–42. doi: 10.1111/jcmm.17217 (PMC8980929; doi:10.1111/jcmm.17217)

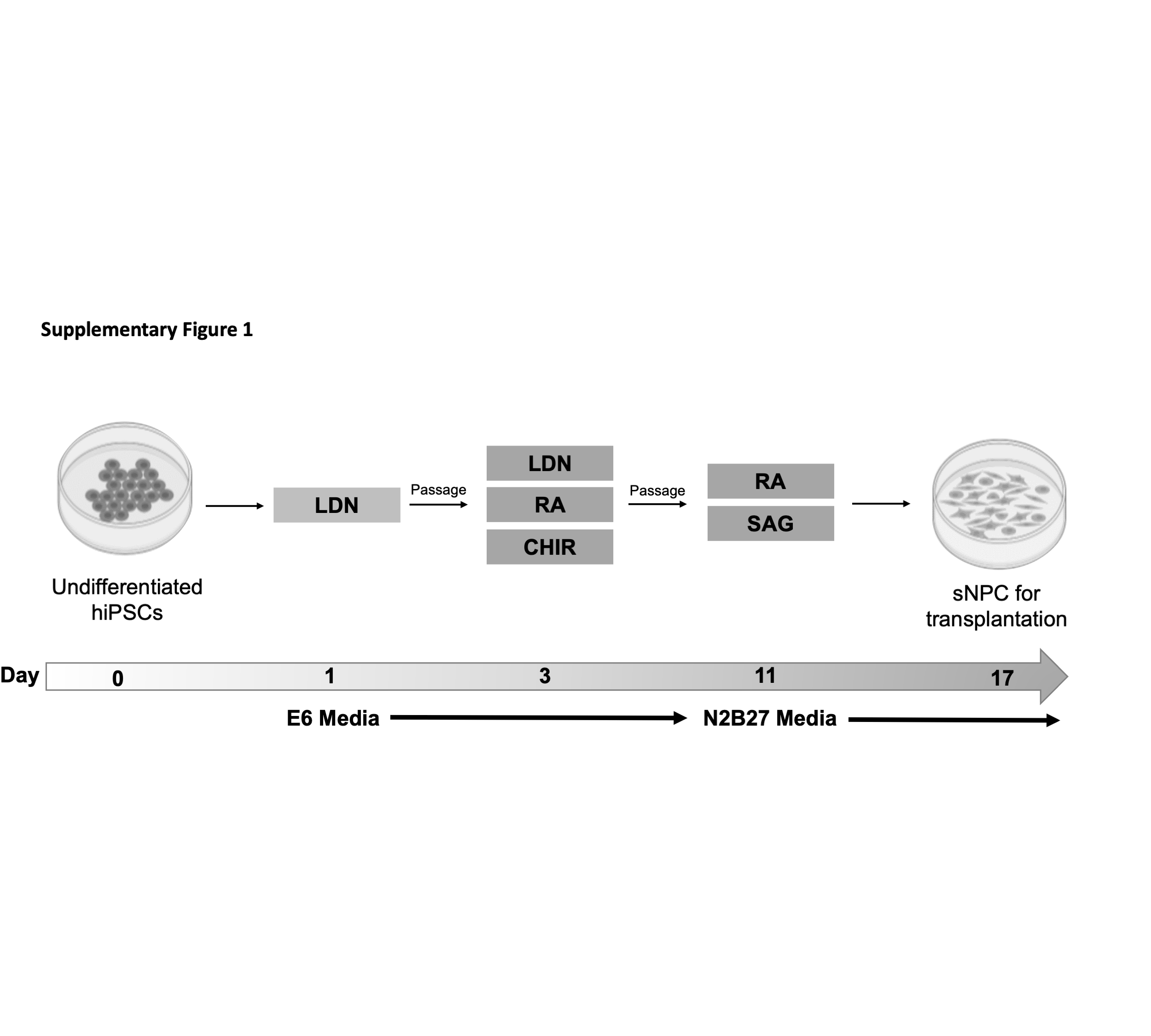

Supplement: Supplementary file 1 — Figure S1 [file JCMM-26-1932-s001.tiff]

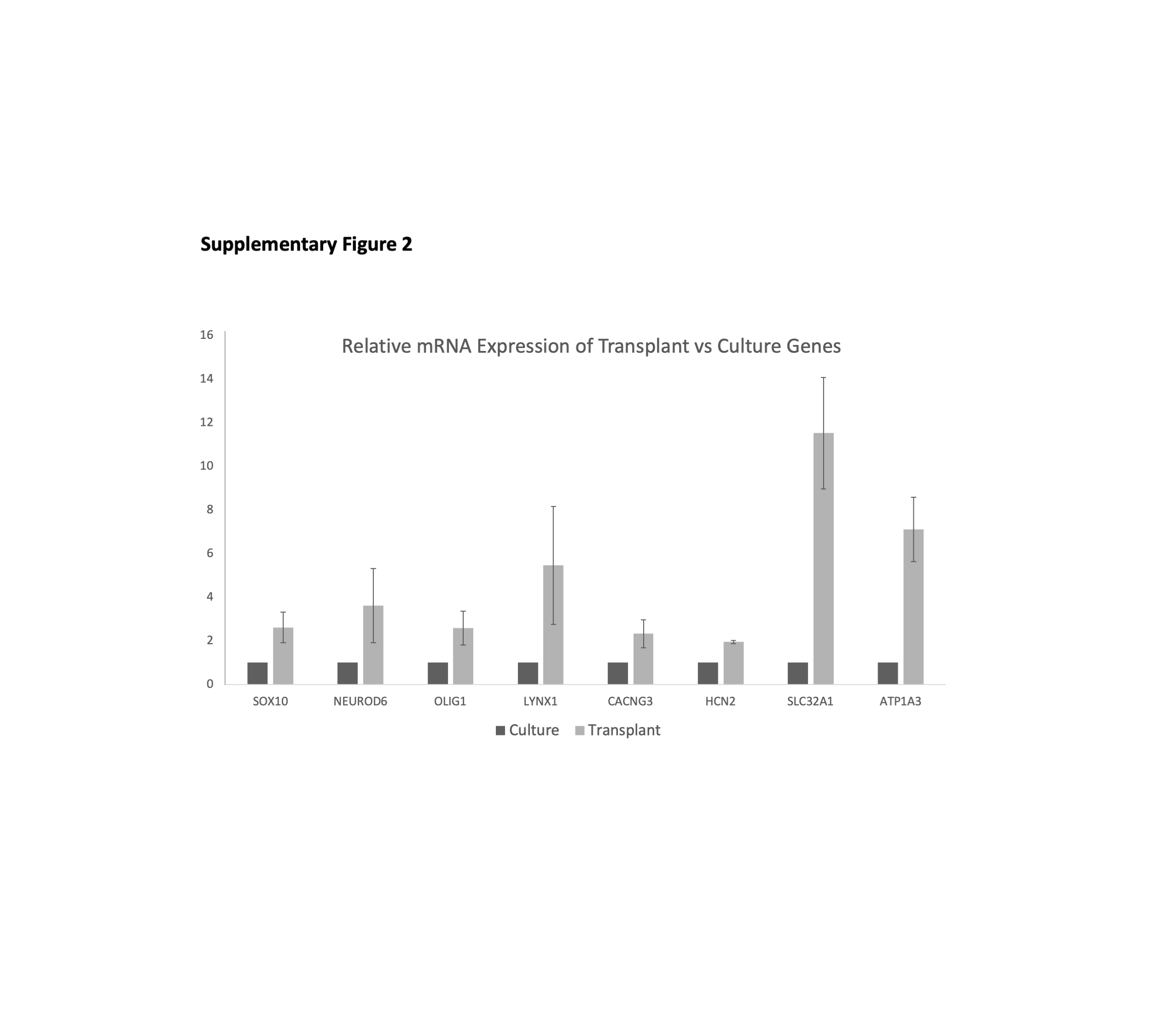

Supplement: Supplementary file 2 — Figure S2 [file JCMM-26-1932-s003.tiff]

## Slide 1
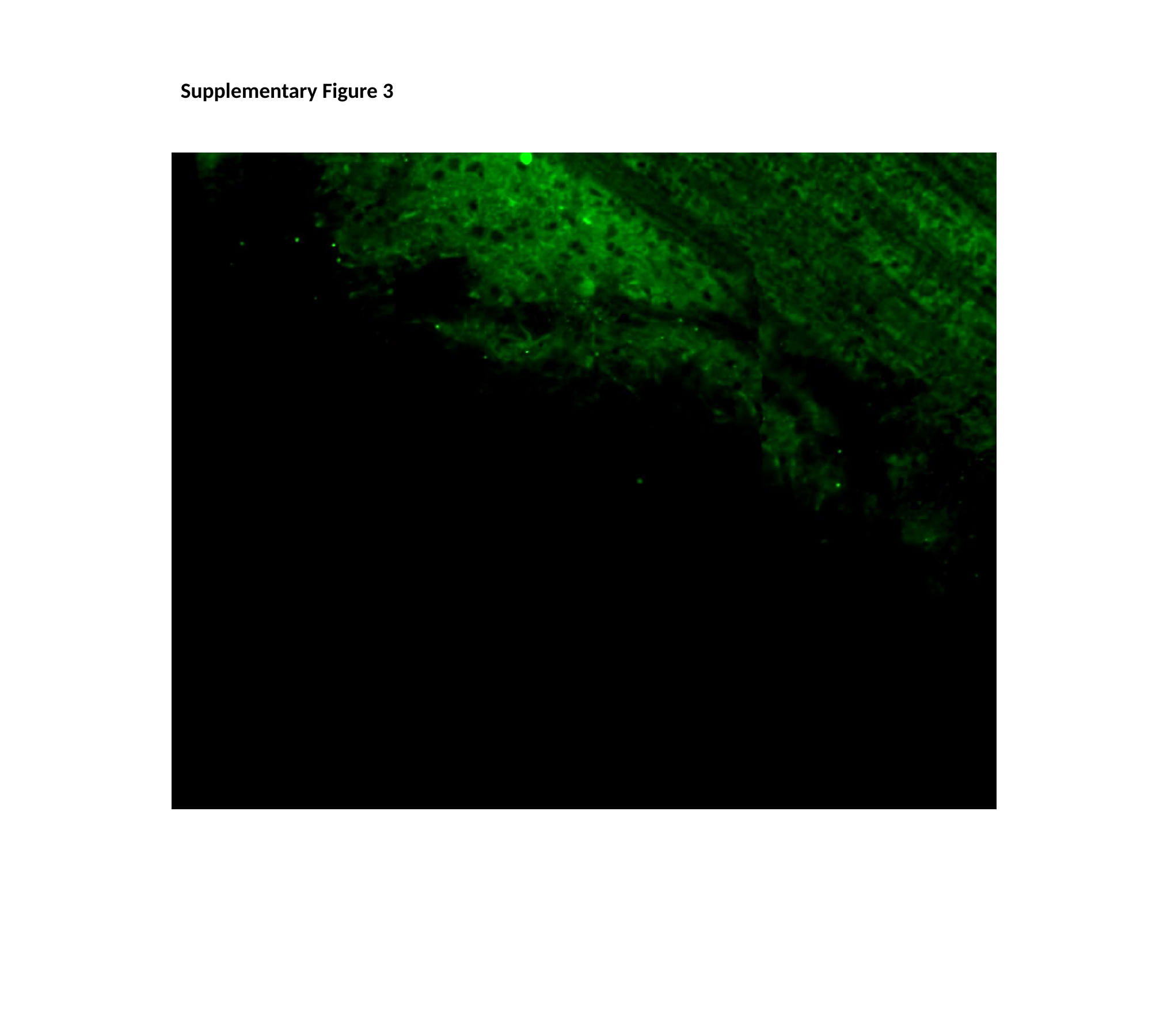

Supplementary Figure 3

Supplement: Supplementary file 3 — Figure S3 [file JCMM-26-1932-s002.pptx]
